# Supplementary material for: Diversity of Legumes in the Cashew Agroforestry System in East Timor (Southeast Asia)
Source: Foods. 2022 Nov 3;11(21):3503. doi: 10.3390/foods11213503 (PMC9655503; doi:10.3390/foods11213503)
Supplement: Supplementary file 1 [file foods-11-03503-s001.zip › foods-1956723-supplementary.pdf]

## *Supplementary Materials:*

# Diversity of legumes in the cashew agroforestry system in East Timor (Southeast Asia)

**Lara Guterres[1,2]†; Maria Cristina Duarte[3]†; Silvia Catarino[4]; Guilherme Roxo[1]; João Barnabé[1]; Mónica Sebastiana[5]; Filipa Monteiro[1,3]; Maria Manuel Romeiras[1,3]***

[1] LEAF—Linking Landscape, Environment, Agriculture and Food Research Center, Associated Laboratory TERRA, Instituto Superior de Agronomia, Universidade de Lisboa, Tapada da Ajuda, 1349-017 Lisboa, Portugal
[2] Faculdade de Ciências Exatas, Universidade Nacional Timor Lorosa'e, Avenida Cidade de Lisboa, Dili, Timor-Leste
[3] cE3c - Center for Ecology, Evolution and Environmental Change & CHANGE - Global Change and Sustainability Institute, Faculdade de Ciências, Universidade de Lisboa, Campo Grande 1749-016 Lisboa, Portugal
[4] Forest Research Center (CEF), Associated Laboratory TERRA, Instituto Superior de Agronomia, Universidade de Lisboa, Tapada da Ajuda, 1349-017 Lisboa, Portugal
[5] BioISI - Biosystems and Integrative Sciences Institute, Faculdade de Ciências, Universidade de Lisboa, Campo Grande 1749-016 Lisboa, Portugal
† The authors contributed equally to this work
*Correspondence: mmromeiras@isa.ulisboa.pt

**Table S1:** Median values (mg/kg wet weight) and standard deviations (SD) of mineral contents of legume seeds from six species (grey rows) and their colour types (white rows) found in East Timor cashew agroforestry system. For species with more than one colour type, the presented values (grey rows) correspond to the median values of all the individual samples. Different letters indicate significant differences among the seed colour types.

| | B | Ca | Cu | Fe | K | Mg | Mn | P | S | Zn |
|---|---|---|---|---|---|---|---|---|---|---|
| ***Cajanus cajan*** | n.q. | **763.541 ± 69.482 a** | **1.947 ± 0.486 a** | **17.158 ± 5.142 a** | **10233.612 ± 383.899 a** | **763.173 ± 48.714 a** | **1.684 ± 0.800 a** | **2130.565 ± 229.839 a** | **1172.175 ± 60.129 a** | **12.760 ± 1.294 a** |
| Black | n.q. | 783.154 ± 47.045 **ab** | 2.196 ± 0.346 **a** | 16.418 ± 2.725 **a** | 10312.38 ± 445.247 **ab** | 779.470 ± 44.328 **ab** | 1.653 ± 0.311 **a** | 2128.167 ± 99.235 **ab** | 1173.505 ± 56.142 **a** | 12.250 ± 1.254 **a** |
| Brownish | n.q. | 723.994 ± 53.587 **c** | 1.612 ± 0.410 **b** | 16.467 ± 5.180 **a** | 10143.8 ± 425.555 **ab** | 747.418 ± 65.300 **ab** | 1.572 ± 0.423 **a** | 2108.035 ± 102.597 **a** | 1133.68 ± 63.431 **a** | 12.066 ± 1.171 **a** |
| Cream | n.q. | 751.462 ± 59.903 **ac** | 2.209 ± 0.498 **a** | 18.292 ± 6.811 **a** | 10025.79 ± 342.415 **a** | 792.826 ± 49.112 **a** | 1.610 ± 0.639 **a** | 2022.974 ± 91.920 **b** | 1219.653 ± 40.269 **b** | 13.865 ± 0.915 **b** |
| Cream-brownish /Purple | n.q. | 788.725 ± 87.850 **b** | 1.593 ± 0.340 **b** | 16.661 ± 4.555 **a** | 10346.264 ± 246.135 **b** | 750.177 ± 22.582 **b** | 1.992 ± 1.156 **b** | 2537.865 ± 90.150 **c** | 1130.144 ± 39.508 **a** | 12.760 ± 1.165 **a** |
| ***Phaseolus lunatus*** | **25.224 ± 5.310 b** | **420.501 ± 70.300 b** | **2.152 ± 2.031 a** | **45.653 ± 7.069 b** | **12770.371 ± 701.176 b** | **1550.581 ± 175.272 b** | **12.488 ± 1.651 b** | **3151.540 ± 632.574 b** | **1108.567 ± 150.129 a** | **15.067 ± 2.779 b** |
| Brownish | 24.308 ± 2.145 **a** | 419.767 ± 42.942 **a** | 2.541 ± 0.285 **a** | 49.892 ± 3.287 **a** | 13228.714 ± 329.229 **a** | 1650.549 ± 65.280 **a** | 14.572 ± 0.722 **a** | 4150.112 ± 178.210 **a** | 1237.106 ± 51.592 **a** | 18.010 ± 0.898 **a** |
| Cream-purple | 24.606 ± 1.643 **a** | 393.313 ± 32.615 **a** | 3.696 ± 0.259 **a** | 51.177 ± 6.056 **a** | 13519.906 ± 223.489 **a** | 1653.021 ± 66.088 **a** | 14.232 ± 0.436 **a** | 4296.713 ± 183.878 **a** | 1496.522 ± 55.818 **a** | 19.805 ± 0.718 **a** |
| Purple-white | 26.484 ± 6.361 **a** | 422.893 ± 80.714 **a** | 1.918 ± 2.455 **b** | 42.873 ± 7.352 **b** | 12391.021 ± 631.721 **b** | 1495.259 ± 167.006 **b** | 12.087 ± 1.245 **b** | 3036.148 ± 248.212 **b** | 1071.721 ± 79.143 **b** | 14.372 ± 2.452 **b** |
| ***Phaseolus vulgaris*** | **11.462 ± 1.299 c** | **858.809 ± 145.896 ac** | **6.853 ± 0.792 b** | **63.326 ± 9.033 c** | **11746.533 ± 1179.656 b** | **1299.074 ± 58.706 c** | **8.990 ± 1.373 c** | **4475.780 ± 289.892 c** | **1618.124 ± 100.161 b** | **27.047 ± 2.669 cd** |
| ***Vigna angularis*** | **5.896** | **2916.046** | **7.622** | **47.600** | **7084.165** | **1777.535** | **21.091** | **2370.304** | **1728.361** | **26.810** |

| | | | | | | | | | | |
|---|---|---|---|---|---|---|---|---|---|---|
| | **± 1.824 d** | **± 62.443 c** | **± 0.653 bc** | **± 4.145 b** | **± 242.509 c** | **± 61.257 d** | **± 5.435 d** | **± 100.535 ad** | **± 72.659 bc** | **± 1.088 cd** |
| ***Vigna radiata*** | **2.528 ± 2.232 d** | **672.647 ± 62.443 d** | **7.803 ± 0.713 bc** | **34.921 ± 5.471 d** | **5604.853 ± 198.382 d** | **1262.969 ± 59.784 c** | **9.804 ± 0.415 c** | **2590.765 ± 63.918 d** | **1660.765 ± 48.119 bc** | **25.588 ± 0.778 c** |
| ***Vigna unguiculata*** | **10.286 ± 4.319 c** | **524.547 ± 69.278 e** | **8.141 ± 0.692 c** | **48.944 ± 7.086 b** | **6954.175 ± 979.536 c** | **1626.304 ± 96.119 b** | **12.977 ± 1.080 b** | **3422.431 ± 167.764 b** | **1862.081 ± 194.575 c** | **29.564 ± 4.395 d** |
| Black | 10.497 ± 2.152 **a** | 484.466 ± 59.145 **a** | 8.251 ± 0.579 **a** | 49.444 ± 9.364 **a** | 6578.416 ± 1219.403 **a** | 1607.647 ± 96.680 **a** | 12.323 ± 1.259 **a** | 3459.091 ± 164.341 **a** | 1990.741 ± 88.192 **a** | 29.348 ± 1.168 **a** |
| Bordeaux | 9.417 ± 5.703 **a** | 552.624 ± 53.036 **b** | 7.975 ± 0.782 **a** | 48.358 ± 3.136 **a** | 7377.762 ± 668.799 **a** | 1671.554 ± 76.952 **b** | 13.166 ± 0.700 **b** | 3407.530 ± 168.864 **a** | 1626.870 ± 135.283 **b** | 29.601 ± 5.731 **a** |

n.q. - not quantified

**Table S2:** Eigenvalues, proportion of variability and variables that contributed to the first four PCs (PCA) based on the mineral content of the different legume species (*Cajanus cajan*, *Phaseolus lunatus*, *P. vulgaris*, *Vigna angularis*, *V. radiata* and *V. unguiculata*).

| Component | PCA1 | PCA2 | PCA3 | PCA4 |
|---|---|---|---|---|
| Eigenvalues | 5,15085762 | 2,57970177 | 1,25946373 | 0,54337349 |
| % Explained variance | 51,5085762 | 25,7970177 | 12,5946373 | 5,4337349 |
| % Cumulative explained variance | 51,50858 | 77,30559 | 89,90023 | 95,33397 |
| Correlation coefficients | | | | |
| K | -0,2042456 | -0,49114524 | 0,1148628 | -0,46110216 |
| Ca | 0,1519788 | 0,29732554 | 0,62524158 | -0,51998907 |
| Mg | 0,3870261 | -0,15450615 | 0,24833578 | 0,3085484 |
| P | 0,2373157 | -0,41496518 | -0,33638731 | -0,37153368 |
| S | 0,3747808 | 0,20935797 | -0,24533918 | -0,01787668 |
| Fe | 0,3702763 | -0,28016801 | -0,04518703 | -0,25946593 |
| Cu | 0,3858888 | 0,2000293 | -0,22687488 | 0,02937041 |
| Zn | 0,3996097 | 0,11889458 | -0,21294956 | -0,13685154 |
| Mn | 0,3578849 | -0,05742644 | 0,48190988 | 0,13496684 |
| B | 0,1217922 | -0,54271108 | 0,17251776 | 0,42226486 |

**Table S3:** Eigenvalues, proportion of variability and variables that contributed to the first four PCs (PCA) based on the mineral content of the different types of *Cajanus cajan*.

| Component | PCA1 | PCA2 | PCA3 | PCA4 |
|---|---|---|---|---|
| Eigenvalues | 2.7252778 | 2.2922557 | 1.399241 | 0.955092 |
| % Explained variance | 30.280864 | 25.469508 | 15.54712 | 10.61214 |
| % Cumulative explained variance | 30.28086 | 55.75037 | 71.29749 | 81.90963 |
| Correlation coefficients | | | | |
| K | 0.18809602 | 0.424218607 | -0.35493427 | 0.3737227 |
| Ca | 0.25281845 | 0.346925792 | -0.12192321 | -0.6586395 |
| Mg | 0.47499067 | -0.068676705 | -0.37914222 | 0.1840965 |
| P | -0.03249576 | 0.603887216 | -0.02130567 | -0.1853891 |
| S | 0.48820578 | -0.141306001 | -0.23834102 | 0.16345 |
| Fe | 0.38172683 | 0.000795477 | 0.50769758 | 0.2950587 |
| Cu | 0.1509504 | -0.440465677 | 0.09526866 | -0.3138744 |
| Zn | 0.48888341 | -0.073606493 | 0.17225706 | -0.3464051 |
| Mn | 0.16786108 | 0.332999794 | 0.60136657 | 0.1611693 |
| B | 0.18809602 | 0.424218607 | -0.35493427 | 0.3737227 |

**Table S4:** Eigenvalues, proportion of variability and variables that contributed to the first four PCs (PCA) based on the mineral content of the different types of *Phaseolus lunatus*.

| Component | PCA1 | PCA2 | PCA3 | PCA4 |
|---|---|---|---|---|
| **Eigenvalues** | 5.41988002 | 1.61145208 | 1.50970507 | 0.716443 |
| **% Explained variance** | 54.1988002 | 16.1145208 | 15.0970507 | 7.164432 |
| **% Cumulative explained variance** | 54.1988 | 70.31332 | 85.41037 | 92.5748 |
| **Correlation coefficients** | | | | |
| **K** | -0.30608717 | 0.45967335 | 0.099701173 | -0.11798947 |
| **Ca** | -0.11131036 | -0.74721292 | 0.004091644 | -0.10608628 |
| **Mg** | -0.33893873 | -0.37211719 | 0.066080438 | -0.37358354 |
| **P** | -0.40060961 | 0.01817444 | -0.015106266 | -0.28919457 |
| **S** | -0.38644279 | 0.22077434 | 0.010668086 | -0.07724913 |
| **Fe** | -0.38468085 | 0.08177945 | 0.105362472 | 0.07705894 |
| **Cu** | -0.17233469 | -0.11976925 | -0.622178153 | 0.523934 |
| **Zn** | -0.37785889 | -0.00111809 | -0.257363146 | 0.32027415 |
| **Mn** | -0.38393072 | -0.07753808 | 0.227308109 | 0.15061395 |
| **B** | -0.03682862 | -0.12563635 | 0.684998272 | 0.58305538 |

**Table S5:** Eigenvalues, proportion of variability and variables that contributed to the first four PCs (PCA) based on the mineral content of the different types of *Vigna unguiculata*.

| Component | PCA1 | PCA2 | PCA3 | PCA4 |
|---|---|---|---|---|
| **Eigenvalues** | 3.81119843 | 1.8982127 | 1.51015692 | 1.14062126 |
| **% Explained variance** | 38.1119843 | 18.982127 | 15.1015692 | 11.4062126 |
| **% Cumulative explained variance** | 38.11198 | 57.09411 | 72.19568 | 83.60189 |
| **Correlation coefficients** | | | | |
| **K** | 0.29136583 | 0.106738872 | -0.19949227 | 0.67331383 |
| **Ca** | -0.34466944 | -0.382042937 | -0.04255544 | 0.26564609 |
| **Mg** | -0.41772232 | -0.266608758 | -0.1456331 | -0.19766343 |
| **P** | -0.31722386 | 0.177123657 | -0.21199346 | -0.42934605 |
| **S** | -0.02624372 | 0.622563804 | 0.31678263 | -0.0542081 |
| **Fe** | -0.07891853 | 0.38945518 | -0.54914554 | -0.01347522 |
| **Cu** | -0.34686594 | 0.42186394 | -0.01133572 | 0.1262259 |
| **Zn** | 0.41960228 | 0.068098906 | -0.30094484 | -0.16885223 |
| **Mn** | -0.2656914 | 0.004512164 | -0.52303242 | 0.27212493 |
| **B** | -0.38379054 | 0.135310589 | 0.3550639 | 0.36201609 |
